# *Zataria multiflora Boiss* and Carvacrol Affect *β*_2_-Adrenoceptors of Guinea Pig Trachea

**DOI:** 10.1155/2011/857124

**Published:** 2010-12-01

**Authors:** Mohammad Hossein Boskabady, Mahsa Kaveh, Naeima Eftekhar, Ali Nemati

**Affiliations:** ^1^Department of Physiology and Pharmaceutical Research Centre, Faculty of Medicine, Mashhad University of Medical Sciences, Mashhad 9177948564, Iran; ^2^Department of Biology, Faculty of Science, Islamic Azad University, Mashhad Branch, Mashhad 918714758, Iran

## Abstract

The stimulatory effect of *Zataria multiflora Boiss* (Labiatae) and carvacrol on *β*-adrenoceptors was examined on guinea pig trachea. The effects of three concentrations of aqueous-ethanolic extract, carvacrol, and propranolol (*β*-receptor antagonist) on *β*-adrenoceptors were tested in nonincubated (group 1, *n* = 8) and incubated tracheal chains with 1 *μ*M chlorpheniramine (histamine H1 receptor antagonist) (group 2, *n* = 5). Isoprenaline (*β*-receptor agonist) curves obtained in the presence of all concentrations of the extract and carvacrol showed leftward shifts compared with that of saline in both groups. In both groups, the EC50 (the effective concentration of isoprenaline, causing 50% of maximum response) obtained in the presence of all concentrations of the extract and carvacrol was significantly lower compared to that of saline (*P* < .01 to *P* < .001). All values of (CR-1: (EC50 in the presence of active substances/EC50 obtained in the presence of saline)-1) obtained in the presence of concentrations of the extract and carvacrol in both groups were negative and significantly different from that of propranolol (*P* < .001 for all cases). The results indicated a stimulatory effect of Zataria multiflora Boiss extract on *β*_2_-adrenoceptors which is perhaps due to its constituent, carvacrol.

## 1. Introduction

*Zataria multiflora Boiss* L is a perennial plant with a woody, fibrous root, and its leaves are small, narrow, and elliptical, greenish-grey in colors. Identified constituents of this plant belong to the classes like terpenes, phenols, aliphatic alcohols, flavonoids, saponins, and tannins. Among the compounds, some classes have been previously identified as bioactive chemicals, particularly terpenes such as thymol and carvacrol. *Zataria multiflora* also contains apigenin, luteolin, and 6-hydroxyluteolin glycosides, as well as di-, tri-, and tetramethoxylated [1, 2].

The extract of this plant has been used to treat coughs due to colds, bronchitis, and pertussis, laryngitis and tonsillitis (as a gargle), the common cold, and disorders of the oral cavity and as an antibacterial agent in oral hygiene by traditional healers in Iran [3–5]. Both the essential oil and thymol are ingredients of a number of proprietary drugs including antiseptic and healing ointments, syrups for the treatment of respiratory disorders, and preparations for inhalation [1]. It has also been used to treat pertussis, stomatitis, and halitosis [2].

Previous studies showed the relaxant effect of this plant in the ileum [6–8] and uterus [9] and another plant of this family (*Thymus vulgaris*) in tracheal smooth muscle [7, 8, 10]. The therapeutic effect of *Zataria* in respiratory disorders of chemical war victims [11], an antitussive effect for the plant [9], and relaxant effect of the plant constituent, carvacrol, on tracheal smooth muscle were also documented [12]. In addition, other effects have been also shown for the plant including antifungal and anticandida effects and effect on different parasites [13–18]. The antibacterial [19–21], anti-inflammatory, analgesic [22–25] and, antinociceptive effects [26] have been also shown for *Zataria multiflora*. 

**Figure 1:**
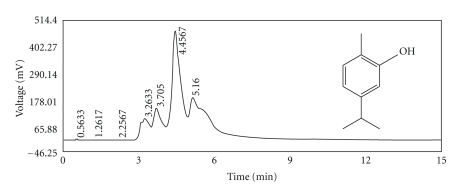
HPLC fingerprint of the the extract of *Z. multiflora* (500 *μ*g/mL) illustrating carvacrol (C10H14O, MW = 150.217) and its chemical structure.

Our previous study showed a potent bronchodilatory effect for carvacrol [12] while our other study did not show any bronchodilatory effect for thymol [27]. Carvacrol is a terpene with phenolic structure. It is a volatile constituent of *Zataria multiflora* essential oil.  Figure 1 illustrates chemical structure of carvacrol, its chemical formula, and molecular weight.

To examine the possible mechanism for the relaxant effect of the plant on smooth muscle, in the present study, the stimulatory effect of aqueous-ethanolic extracts of *Zataria multiflora Boiss*, and its constituent, carvacrol on *β*-adrenoceptors was examined on tracheal chains of guinea pigs. In fact, if *Zataria multiflora Boiss* and its constituents have stimulatory effect on *β*-adrenoceptors, they could have a potential therapeutic effect on obstructive pulmonary diseases such as asthma and chronic obstructive pulmonary diseases (COPD).

## 2. Material and Methods

### 2.1. Plant and Extracts

*Zataria multiflora Boiss* was collected From a mountain in the region between Tabas and Yazd, (centre east region of Iran), Fleurine mine, and identified by M. R. Joharghi. A voucher specimen was preserved in the Herbarium of the School of Agriculture, Ferdowsi University (Herbarium no: 35314, FUMH). The aqueous-ethanolic extract of the plant was prepared as follows: fifty grams of *Zataria multiflora* seeds were grinded and added to 700 mL of ethanol 50% (350 mL distilled water and 350 mL ethanol) using the Soxhlet apparatus. The solvent was then removed under reduced pressure. The extract concentration in the final extract was adjusted to 0.1 g/mL by adding distilled water to the dried extract.

### 2.2. Characterization of the Extract of Zataria multiflora by HPLC

The quality of the extract of *Zataria multiflora* was characterized by HPLC (Waters 474, Waters Corporation, MA, USA) fingerprint. The extract was dissolved in mobile phase and filtered through 0.22 *μ*m membrane filter. An aliquot (20 *μ*L) of sample (500 *μ*g/mL) was injected to the reverse phase HPLC column (C18). The mobile phase consisted of phosphate buffer (pH = 4.8) : methanol : acetonitrile (40 : 30 : 30) with an isocratic elution at the flow rate of 1 mL/minute. The peaks were monitored at 280 nm (Figure 1). All solvents used were HPLC grade and supplied by Caledon Laboratories, Georgetown Ltd, Canada.

### 2.3. Tissue Preparations

Male Dunkin-Hartley guinea pigs (400–700 g) were sacrificed by a blow on the neck, and the tracheas were removed. Each trachea was cut into 10 rings (each containing 2-3 cartilaginous rings). All the rings were then cut open opposite the trachealis muscle and sutured together to form tracheal chain [28]. Tissue was then suspended in a 10 mL organ bath (organ bath 61300, Bio Science Palmer-Washington, Sheerness, Kent UK) containing Krebs-Henseleit solution with the following composition (mM): NaCl 120, NaHCO_3_ 25, MgSO_4_ 0.5, KH_2_PO_4_ 1.2, KCl 4.72, CaCl_2_ 2.5, and dextrose 11.

Krebs solution was maintained at 37°C and gassed with 95% O_2_ and 5% CO_2_. Tissue was suspended under isotonic tension (1 g) and allowed to equilibrate for at least 1 hr while it was washed with Krebs solution every 15 min. This study was approved by the University's Ethics Committee. The allowance number of the relevant ethical committee for the animal experiments is 85301.

### 2.4. Protocols

The stimulatory effect of of *Zataria multiflora Boiss* and carvacrol on *β*_2_-adrenoceptors was examined by producing the cumulative log concentration-response curve of isoprenaline sulphate-induced (Sigma Chemical Ltd., UK) relaxation of precontracted tracheal chains by 10 *μ*m methacholine hydrochloride (Sigma Chemical Ltd., UK) 10 min after the exposure of tissue to one solution. Different tested solutions were included: 10 nM propranolol (0.1 mL of propranolol hydrochloride with 0.1 *μ*m concentration, Sigma Chemical Ltd., UK), three concentrations of aqueous-ethanolic extract from *Zataria multiflora Boiss* (0.5, 1, and 2 *μ*g/mL), and carvacrol (Fluka, Italy, Catalogue no. C4915, purity 75%) (0.1, 0.2, and 0.4 *μ*g/mL) or 0.2 mL saline. The consecutive concentrations of isoprenaline were added every 2 min (including 5 nM–1000 *μ*m); and the percentage of relaxation due to each concentration in proportion to the maximum relaxation obtained in the presence of saline was plotted against log concentration of isoprenaline. The effective concentration of isoprenaline causing 50% of maximum response (EC_50_) in each experiment was measured using the log concentration-response curve of the corresponding experiment.

The shift of cumulative log concentration-response curves obtained in the presence of different concentrations of extract, carvacrol, and propranolol was examined by comparing the EC_50_ obtained in the presence of each solution with that of saline. In addition, the maximum responses to isoprenaline obtained in the presence of different concentrations of extract, carvacrol, and propranolol in all sets of experiments were compared with that of saline. To examine the parallel rightward shift, the slope of the isoprenaline-response curve of each experiment was measured and was compared with that of saline. In experiments with parallel shift in isoprenaline-response curve, the concentration-ratio minus one (CR-1) as an index of the competitive antagonism effect was calculated by the following equation:

(1) $$\left(\frac{{\text{EC}}_{50}\;\text{obtained}\;\text{in}\;\text{the}\;\text{presence}\;\text{of}\;\text{effective}\;\text{solutions}}{{\text{EC}}_{50}\;\text{obtained}\;\text{in}\;\text{the}\;\text{presence}\;\text{of}\;\text{saline}\;}\right)-1.$$

The stimulatory effect of *Zataria multiflora* on *β*_2_-adrenoceptors was tested on two different experimental conditions as follows:

- nonincubated tracheal chains (group 1, *n* = 8),
- incubated tracheal chains 30 min prior to the beginning and while obtaining the isoprenaline curve with 1 *μ*m chlorpheniramine maleate (Sigma Chemical Ltd., UK) (group 2, *n* = 5).

Isoprenaline is *β*-receptor agonist which causes relaxation of airway smooth muscle. Propranolol is a *β*-receptor competitive antagonist, and chlorpheniramine is a histamine H_1_ receptor competitive antagonist. Competitive antagonists bind to their specific receptor and prevent agonist binding to the receptor, causing a parallel shift in agonist concentration-response curve.

All of the experiments were performed randomly with 1-hour resting period of tracheal chains between each two experiments while washing the tissues every 15 min with Krebs solution. In all experiments contractions were measured using an isotonic transducer (Harvard APP LTD, 50-6360 SINO 0210) and measured using a software by computer (Acer model no.: G781) recording.

### 2.5. Statistical Analysis

All data were expressed as mean ± SEM. The EC_50_, slope, and maximum response obtained in the presence of extract, carvacrol, and propranolol were compared with those obtained in the presence of saline and (CR-1) obtained in the presence of extract and carvacrol, with those obtained in the presence of propranolol using the paired *t*-test. The comparison of the data of different concentrations of extract and carvacrol was performed using one-way analysis of variance (ANOVA) with Tukey-Kramer multiple posttest. The values of EC_50_, the slope, (CR-1), and maximum response obtained in group 2 experiments were compared with those of group 1 using unpaired “*t*” test. Correlations between the concentrations of the extract and carvacrol with the values of EC_50_ and (CR-1) were examined using least square regression. Significance was accepted at *P* < .05.

## 3. Results

### 3.1. Characterization of the Extract

Carvacrol of the extract from *Zataria multiflora* was identified using HPLC method in the Department of Pharmacology, Medical School, Mashhad University of Medical Sciences (Figure 1).

### 3.2. The Extract and Carvacrol Stimulate *β*-Adrenoceptors

**Figure 2:**
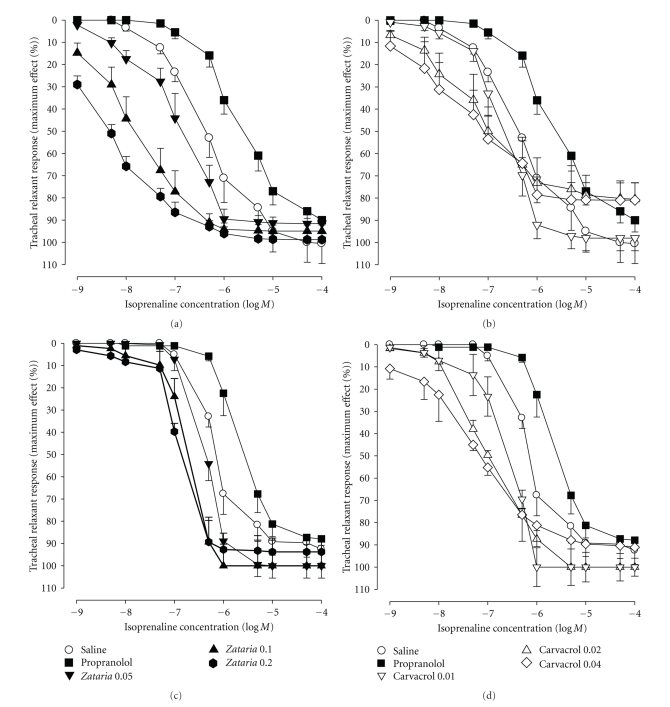
Cumulative log concentration-response curves of isoprenaline-induced relaxation of guinea pig tracheal chains, in the presence of saline, three concentrations of aqueous-ethanolic extract, three concentrations of carvacrol, and 10 nM propranolol in nonincubated trachea ((a) and (b): *n* = 8) and incubated tissues with chlorpheniramine ((c) and (d): *n* = 5).

Cumulative log concentration-response curves to isoprenaline obtained in the presence of all concentrations of the extract and carvacrol showed clear leftward shift while the curve of propranolol showed clear rightward shift compared to isoprenaline curves produced in the presence of saline in both groups 1 and 2 (Figures 2(a)–2(d)).

**Figure 3:**
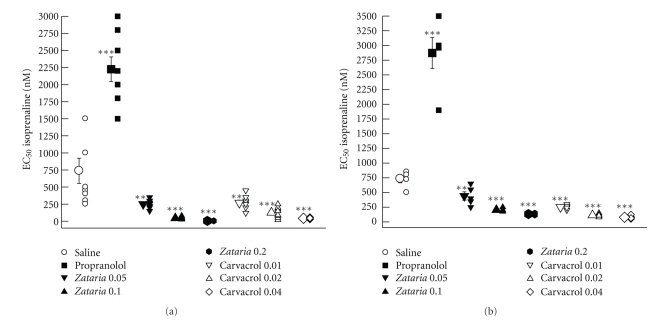
Isoprenaline EC_50_ obtained in the presence of three concentrations of aqueous-ethanolic extract from *Z. multiflora* (0.5 Zataria 0.05, 1 Zataria 0.1, and 2 *μ*g/mL, Zataria 0.2), carvacrol (0.1 Carvacrol 0.01, 0.2 Carvacrol 0.02, and 0.4 *μ*g/mL, Carvacrol 0.04), 10 nM propranolol (Propranolol), and saline (O) in nonincubated trachea (a) (group 1: *n* = 8) and (b) incubated tissues with chlorpheniramine (group 2: *n* = 5). Statistical comparison between saline and other solutions. NS: nonsignificant difference, **P < .01, ***P < .001. Statistical comparison between different concentrations of the extract and carvacrol ^+^ *P* < .01, ^+++^ *P* < .001. EC_50_ obtained in the presence of three concentrations of the extract in group 1 (*P* < .05 for low concentration and *P* < .001 for two higher concentrations) and only high concentration of carvacrol (*P* < .01) was higher than group 2. The comparison of EC_50_ obtained in the presence of extract, carvacrol, and propranolol with that of saline and the data between two groups was done using the paired *t*-test. The comparison of the data of different concentrations of extract and carvacrol was performed using one-way analysis of variance (ANOVA) with Tukey-Kramer multiple post test.

Isoprenaline EC_50_ obtained in the presence of propranolol was significantly higher than that of saline in both groups of experiments (*P* < .001). However, the EC_50_ obtained in the presence of all concentrations of the extract and carvacrol was significantly lower than that of saline in both groups 1 and 2 (*P* < .01 to *P* < .001), (Figures 3(a) and 3(b)). 

**Figure 4:**
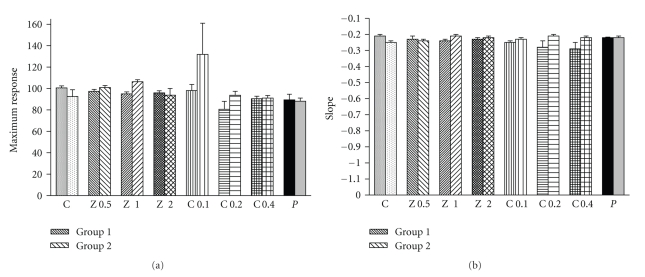
Values of maximum response to isoprenaline (a) and slope of isoprenaline log concentration-response curves (b) obtained in the presence of different concentrations of extract from *Zataria multiflora*, carvacrol, 10 nM propranolol, and saline in nonincubated trachea (fine filled bars) (group 1: *n* = 8) and incubated tissues with chlorpheniramine (medium filled bars) (group 2: *n* = 5). There was not significant difference between the data of the extract and carvacrol with those of saline and between the data of two groups and the data of the extract with those of carvacrol. The comparison of slope and maximum response obtained in the presence of extract, carvacrol, and propranolol with those of saline and data of two groups were done using the paired *t*-test. The comparison of the data of different concentrations of extract and carvacrol was performed using one-way analysis of variance (ANOVA) with Tukey-Kramer multiple post test.

Maximum responses to isoprenaline obtained in the presence of all concentrations of the extract and carvacrol were not significantly different compared to that of saline in both groups 1 and 2 (Figure 4(a)). Slopes of isoprenaline-response curves obtained in the presence of all three concentrations of the extract and carvacrol were not significantly different from that of saline in both groups 1 and 2 (Figure 4(b)).

**Figure 5:**
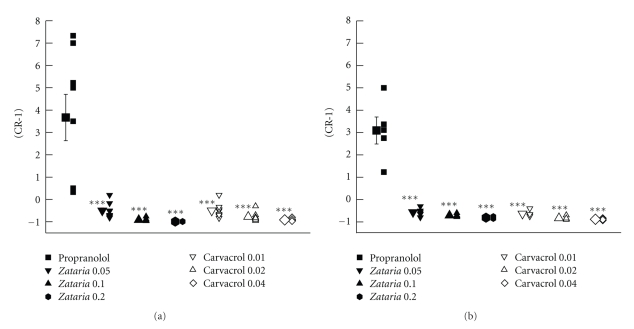
Values of (CR-1) obtained in the presence of three concentrations of aqueous-ethanolic extract from *Zataria multiflora* (0.5 Zataria 0.05, 1 Zataria 0.1, and 2 *μ*g/mL, Zataria 0.2), carvacrol (0.1 Carvacrol 0.01, 0.2 Carvacrol 0.02, and 0.4 *μ*g/mL, Carvacrol 0.04), and 10 nM propranolol (Propranolol) in nonincubated trachea (a) (group 1: *n* = 8) and (b) incubated tissues with chlorpheniramine (group 2: *n* = 5). Statistical comparison between chlorpheniramine and other solutions. NS: nonsignificant difference, ***P < .001. Statistical comparison between different concentrations of the extract and carvacrol ^+^ *P* < .05, ^++^ *P* < .01. Values of (CR-1) obtained in the presence of two higher concentrations of the extract in group 1 were higher than those in group 2 (*P* < .001 for two higher concentrations). The comparison of (CR-1) obtained in the presence of extract and carvacrol with those of propranolol and data between two groups was done using the paired *t*-test. The comparison of the data of different concentrations of extract and carvacrol was performed using one-way analysis of variance (ANOVA) with Tukey-Kramer multiple post test.

Values of (CR-1) obtained in the presence of all concentrations of the extract and carvacrol were negative and significantly different from those of propranolol in both groups 1 and 2 (*P* < .001 for all cases) (Figures 5(a) and 5(b)). There were significant negative correlations between the concentrations of the extract and carvacrol with the values of EC_50_ and (CR-1) in both groups (*P* < .005 to *P* < .001) (Table 1).

### 3.3. Differences between the Extract of Zataria multiflora and Carvacrol

Values of isoprenaline EC_50_ obtained in the presence of two higher concentrations of the extract were significantly lower than those of carvacrol in group 1 (*P* < .05 to *P* < .001) (Figure 3(a)). The values of (CR-1) obtained in the presence of two higher concentrations of the extract were significantly greater than those of carvacrol in group 1 (*P* < .05 to *P* < .01) (Figure 5(a)). In group 2 experiment, the values of isoprenaline EC_50_ obtained in the presence of all concentrations of the extract were significantly greater than those of carvacrol (*P* < .05 to *P* < .001) (Figure 3(b)). The values of (CR-1) obtained in the presence of two higher concentrations of the extract were significantly lower than those of carvacrol (*P* < .05 for both cases) (Figure 5(b)). However, there was no significant difference in maximum response to isoprenaline and slope between the extract and carvacrol in both groups (Figures 4(a) and 4(b)).

Values of isoprenaline EC_50_ obtained in the presence of all concentrations of the extract and only in the highest concentration of carvacrol in group 1 were significantly lower than those in group 2 (*P* < .05 to *P* < .001) (Figure 3). The values of (CR-1) obtained in the presence of two higher concentrations of the extract in group 1 were significantly greater than those of group 2 (*P* < .001 for both cases) (Figure 5). However, there was no significant difference in maximum response to isoprenaline and slope between the two groups (Figures 4(a) and 4(b)).

## 4. Discussion

In the present study the stimulatory effect of the aqueous-ethanolic extract of the plant on *β*-adrenoceptors (as one possible mechanism responsible for the observed relaxant effect seen for the extract of *Zataria multiflora Boiss* and its constituent, carvacrol) on tracheal and other smooth muscles [6–10] was examined. In fact, the relaxant effect of tracheal smooth muscle due to stimulation of *β*-adrenoceptors is a well-known phenomenon [29]. The parallel rightward shifts in isoprenaline log concentration-response curves obtained in the presence of the different concentrations of aqueous-ethanolic extract and carvacrol and the achievement of maximum relaxation effect to isoprenaline compared to that of saline showed possible stimulatory effects of the hydroethanolic extract of the plant and carvacrol on *β*-adrenoceptors of guinea pig's trachea [29–31]. Lower values of EC_50_ obtained in the presence of the different concentrations of aqueous-ethanolic extract and carvacrol compared to that of saline confirmed this effect. In addition the negative values of (CR-1) obtained in the presence of the extract and carvacrol are another indicator of stimulatory effect of the plant and its constituent on *β*-adrenoceptors of trachea smooth muscle.

The stimulatory effect of extract from *Zataria multiflora* and carvacrol on *β*_2_-adrenoceptors was also examined on incubated tracheal preparation with chlorpheniramine to block histamine H_1_ receptors, (group 2 experiments). The group 2 experiment was done in order to evaluate the effect of the extract and carvacrol on *β*_2_-adrenoceptors more precisely. The results of group 2 experiments were very similar to those obtained in group 1, confirming the stimulatory effect of the extract and carvacrol on *β*_2_-adrenoceptors [29–31]. The dose response curves obtained in the presence of extract in group 2 (incubated with chlorpheniramine) showed smaller leftward shift compared to group 1, but for carvacrol the shift in the two groups was similar. In addition the values of EC_50_ obtained in the presence of concentrations of extract in group 1 were significantly lower than those of group 2. These results indicated an inhibitory effect for the extract on histamine (H1) receptors in addition to stimulatory effect of the plant on *β*_2_-adrenoceptors.

The significant negative correlations between both values of EC_50_ and (CR-1) and the concentrations of the extract and carvacrol indicated their concentration-dependent stimulatory effect on *β*_2_-adrenoceptors. However, the significant lower values of EC_50_ isoprenaline obtained in the presence of all concentrations of the extract in group 1 compared to those of group 2 and the higher values of (CR-1) obtained in the presence of two higher concentrations of the extract in group 1 may indicate an inhibitory effect of the extract on histamine (H_1_) receptors in addition to its stimulatory effect on *β*_2_-adrenoceptors. For carvacrol, the values of EC_50_ isoprenaline obtained in the presence of only the highest concentration of carvacrol in group 1 were significantly lower than those in group 2, and there was not significant difference in the values of (CR-1) obtained in the presence of concentrations of carvacrol between the two groups which is an indicator of the absence of inhibitory effect of carvacrol on histamine (H_1_) receptors. In fact, in our previous study the relaxant effect of macerated and aqueous extract of other species plant from this family was almost completely abolished in incubated tracheal preparations incubated with propranolol and chlorpheniramine [10] which confirms the result of the present study. However, the effect of both the extract and carvacrol on histamine (H_1_) receptors should be examined in further studies more precisely. 

In addition, the values of EC_50_ isoprenaline obtained in the presence of two higher concentrations of the extract in group 1 were significantly lower and those of its all concentrations in group 2 were greater than those of carvacrol. The values of (CR-1) obtained in the presence of two higher concentrations of the extract in group 1 were significantly greater and in group 2 were lower than those of carvacrol. These results also support the suggestion of an inhibitory effect on histamine (H_1_) receptors for the extract in addition to its stimulatory effect on *β*_2_-adrenoceptors. These results indicated the differences between the effects of hydroethanolic extract of *Zataria multiflora* and its constituent carvacrol. Therefore, the pharmacological properties of the extract are not solely due to its constituent, carvacrol, which is a very important finding of this study; because in phytotherapy it is the extract that defines the efficacy not some analytical lead substance, even if it contributes to the overall effect.

**Figure 6:**
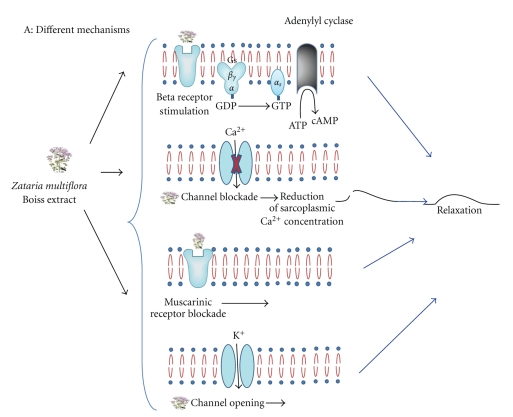
Suggested mechanism for the relaxant effect of the extract from *Zataria multiflora* on tracheal smooth muscle (A) including (a) beta-adrenergic stimulatory effect, (b) calcium channels opening effect, (c) muscarinic inhibitory effect, and (d) potassium channel opening effect.

The concentration of carvacrol in the essential oil of* Zataria multiflora* is reported between 17% and 40%, with a mean value of 30% [32–34] which should be lesser in the hydroalcoholic extract. Therefore, the concentrations of carvacrol used in the present study are equal to those of extract (the concentrations of carvacrol were one fifth of the extract). Thus the stimulatory effect of the extract of *Zataria multiflora* on *β*_2_-adrenoceptors could be due to its constituent carvacrol. Different suggested mechanism for the relaxant effect of the extract from *Zataria multiflora* on tracheal smooth muscle and the contribution of its constituent carvacrol was illustrated in Figures 5 and 6. The major limitation of the present study is that the selectivity of the stimulatory effect of *Zataria multiflora Boiss* and its constituent carvacrol on *β*_2_-adrenoceptors can not estimated from the present results precisely, and further studies needed to find out their selectivity on these receptors. In addition, the bronchodilatory and *β*_2_-receptor stimulatory effect should be examined in further studies.

## 5. Conclusions

In conclusion, these results indicated a relatively potent stimulatory effect for the extract from *Zataria multiflora Boiss* on *β*_2_-adrenoceptors which is perhaps due to its constituent, carvacrol. A possible inhibitory effect of the plant on histamine (H_1_) receptors was also suggested.

## Figures and Tables

**Figure 7:**
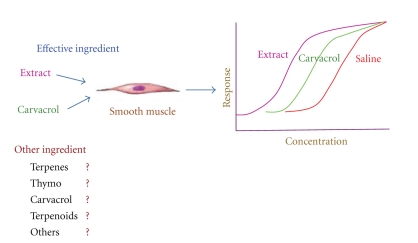
The contribution of carvacrol and suggested contribution of other constituents of the plant on *β*-adrenoceptors stimulatory effect of the plant (shifting concentration response curve of isoprenaline to the left).

**Table 1:** Correlation (*r*) between EC_50_ isoprenaline and (CR-1) with concentrations of the extract and carvacrol in two different experimental groups.

Solutions	Group 1				Group 2			
	EC_50_		(CR-1)		EC_50_		(CR-1)	
	*R*	*P*	*R*	*P*	*R*	*P*	*r*	*P*
Extract	−0.804	*P* < .001	−0.625	*P* < .001	−0.742	*P* < .001	−0.661	*P* < .001
Carvacrol	−0.755	*P* < .001	−0.590	*P* < .005	−0.810	*P* < .001	−0.688	*P* < .001

## References

[1] SCOP (1997). *“Thymi herba” Monographs on the Medicinal Uses of Plant Drugs*.

[2] Mossa JS, Al-Yahya MA, Al-Meshal IA (1987). *Medicinal Plants of Saudi Arabia*.

[3] Mozzaffarian V (1998). *A Dictionary of Iranian Plant Names*.

[4] Sharaf-Kandi AR, Avicenna (1985). *The Canon of Medicine*.

[5] Zargari A (1990). *Medical Plants*.

[6] Gharib Naseri MK (2003). Effect of *Zataria multiflora Boiss* leaf hydroalchoholic extract on rat ileum. *Behbood Journal*.

[7] Stecher PG (1968). *The Merck Index: An Encyclopedia of Chemicals and Drugs*.

[8] Reiter M, Brandt W (1985). Relaxant effects on tracheal and ileal smooth muscles of the guinea pig. *Arzneimittelforschung*.

[9] Gharib Naseri MK, Mazlomi H, Goshaiesh M, Vakilzadeh G, Heidari A (2006). Antispasmodic effect of *Zataria multiflora Boiss*. Leaf extract on the rat uterus. *Iranian Journal of Pharmaceutical Research*.

[10] Boskabady MH, Aslani MR, Kiani S (2006). Relaxant effects of *Tymus volgaris* on guinea pig tracheal chains and its possible mechanism(s). *Phytotherapy Research*.

[11] Mostafavi B, Shasavari S (2005). The effect of *Zataria* on respiratory disorders of chemical war victims.. *Behbood Journal*.

[12] Boskabady MH, Jandaghi P (2003). Relaxant effects of carvacrol on guinea pig tracheal chains and its possible mechanisms. *Pharmazie*.

[13] Llewellyn GC, Burkett ML, Eadie T (1981). Potential mold growth, aflatoxin production, and antimycotic activity of selected natural spices and herbs. *Journal of the Association of Official Analytical Chemists*.

[14] Jafari S, Amanlou M, Borhan-Mojabi K, Farsam H (2003). Comparative study of *Zataria multiflora* and *Anthemis nobelis* extracts with *Myrthus communis* preparation in the treatment of recurrent aphthous stomatitis. *Daru*.

[15] Azadbakht M, Ziaei H, Abdollahi F, Shabankhani B (2003). Effect of essential oils of *Artemisia*, *Zataria* and *Myrtus commonis* on Trichomonas vaginalis. *Journal of Medicinal Plant*.

[16] Khosravi AR, Eslami AR, Shokri H, Kashanian M (2008). *Zataria multiflora* cream for the treatment of acute vaginal candidiasis. *International Journal of Gynecology and Obstetrics*.

[17] Mahmoudabadi AZ, Dabbagh MA, Fouladi Z (2007). In vitro anti-Candida activity of *Zataria multiflora Boiss*. *Evidence-Based Complementary and Alternative Medicine*.

[18] Vukovic N, Milosevic T, Sukdolak S, Solujic S (2007). Antimicrobial activities of essential oil and methanol extract of Teucrium montanum. *Evidence-Based Complementary and Alternative Medicine*.

[19] Shafiee A, Javidnia K, Tabatabai M (1999). Volatile constituents and antimicrobial activity of *Zataria multiflora*, population Iran. *Iranian Journal of Chemistry and Chemical Engineering*.

[20] Janssen AM, Scheffer JJC, Baerheim Svendsen A (1987). Antimicrobial activity of essential oils: a 1976–1986 literature review. Aspects of the test methods. *Planta Medica*.

[21] Simbar M, Azarbad Z, Mojab F, Alavi Majd H (2008). A comparative study of the therapeutic effects of the *Zataria multiflora* vaginal cream and metronidazole vaginal gel on bacterial vaginosis. *Phytomedicine*.

[22] Amanlou M, Beitollahi JM, Abdollahzadeh S, Tohidast-Ekrad Z (2006). Miconazole gel compared with *Zataria multiflora Boiss*. Gel in the treatment of denture stomatitis. *Phytotherapy Research*.

[23] Nakhai LA, Mohammadirad A, Yasa N (2007). Benefits of *Zataria multiflora Boiss* in experimental model of mouse inflammatory bowel disease. *Evidence-Based Complementary and Alternative Medicine*.

[24] Malihezaman M, Mahbobe M, Maryam SL (2007). The ultrastructural and stereological study of aqueous extracts of *Zataria multiflora Boiss* and Elaeagnus angustifolia on the mouse fetus stomach. *Journal of Biological Sciences*.

[25] Hosseinzadeah H, Salmani G (2003). Antiocceptive, anti-inflammatory and acute toxity effects of *Zataria multiflora BoissS* extracts in mic and rat. *Journal of Ethnopharmacology*.

[26] Ramezani M, Hosseinzadeh H, Samizadeh S (2004). Antinociceptive effects of *Zataria multiflora Boiss* fractions in mice. *Journal of Ethnopharmacology*.

[27] Boskabady MH, Rakhshandah H, Moetamedshariati V (1998). Bronchodilatory and anticholinergic effects of *Carum copticum* on isolated guinea pig tracheal chains. *Medical Journal of The Islamic Republic of Iran*.

[28] Holroyde MC (1986). The influence of epithelium on the responsiveness of guinea-pig isolated trachea. *British Journal of Pharmacology*.

[29] Linden A, Bergendal A, Ullman A, Skoogh B-E, Lofdahl C-G (1993). Salmeterol, formoterol, and salbutamol in the isolated guinea pig trachea: differences in maximum relaxant effect and potency but not in functional antagonism. *Thorax*.

[30] Arunlakshana O, Schild HO (1959). Some quantitive use of drug antagonist. *British Journal of Pharmacology*.

[31] Ariens EJ, Nadel JA, Pauwels R, Snashall PD (1987). Pharmacology of the airway smooth muscle. *Bronchial Hyperresponsiveness*.

[32] Mahboubi M, Feizabadi MM, Safarab M (2008). Antifungal activity of essential oils from *Zataria multiflora*, Rosmarinus officinalis, Lavandula stoechas, Artemisia sieberi Besser and Pelargonium graveolens against clinical isolates of Candida albicans. *Pharmacognosy Magazine*.

[33] Abkenar SD, Yamini Y, Shemirani F, Assadi Y (2008). Headspace solid phase microextraction using a porous-layer activated charcoal coating fused silica fiber for identification of volatile organic compounds emitted by *Zataria multiflora Boiss*. *Chemia Analityczna*.

[34] Sharififar F, Moshafi MH, Mansouri SH, Khodashenas M, Khoshnoodi M (2007). In vitro evaluation of antibacterial and antioxidant activities of the essential oil and methanol extract of endemic *Zataria multiflora Boiss*. *Food Control*.

